# Association between arterial hypertension and liver outcomes using polygenic risk scores: a population-based study

**DOI:** 10.1038/s41598-022-20084-z

**Published:** 2022-09-16

**Authors:** Fredrik Åberg, Katri Kantojärvi, Ville Männistö, Anna But, Veikko Salomaa, Teemu Niiranen, Martti Färkkilä, Panu Luukkonen, Satu Männistö, Annamari Lundqvist, Markus Perola, Antti Jula

**Affiliations:** 1 Transplantation and Liver Surgery Clinic, Helsinki University Hospital, Helsinki University, Helsinki, Finland; 2 Finnish Institute for Health and Welfare, Helsinki, Finland; 3 Departments of Medicine, Kuopio University Hospital, University of Eastern Finland, Kuopio, Finland; 4 Biostatistics Consulting, Department of Public Health, Helsinki University Hospital, University of Helsinki, Helsinki, Finland; 5 Department of Medicine, Turku University Hospital, University of Turku, Turku, Finland; 6 Abdominal Center, Helsinki University Hospital, Helsinki University, Helsinki, Finland; 7 Minerva Foundation Institute for Medical Research, Helsinki, Finland; 8 Department of Medicine, Helsinki University Hospital, University of Helsinki, Helsinki, Finland; 9 Department of Internal Medicine, Yale University, New Haven, CT USA; 10 HUCH Meilahti Hospital, HUS, PB 372, 00029 Helsinki, Finland

**Keywords:** Hepatology, Epidemiology

## Abstract

Arterial hypertension (HTA) is associated with liver disease, but causality remains unclear. We investigated whether genetic predisposition to HTA is associated with liver disease in the population, and if antihypertensive medication modifies this association. Participants of the Finnish health-examination surveys, FINRISK 1992–2012 and Health 2000 (n = 33,770), were linked with national electronic healthcare registers for liver-related outcomes (K70-K77, C22.0) and with the drug reimbursement registry for new initiation of antihypertensive medication during follow-up. Genetic predisposition to HTA was defined by polygenic risk scores (PRSs). During a median 12.9-year follow-up (409,268.9 person-years), 441 liver-related outcomes occurred. In the fully-adjusted Cox-regression models, both measured systolic blood pressure and clinically defined HTA were associated with liver-related outcomes. PRSs for systolic and diastolic blood pressure were significantly associated with liver-related outcomes (HR/SD 1.19, 95% CI 1.01–1.24, and 1.12, 95% CI 1.01–1.25, respectively). In the highest quintile of the systolic blood pressure PRS, new initiation of antihypertensive medication was associated with reduced rates of liver-related outcomes (HR 0.55, 95% CI 0.31–0.97). HTA and a genetic predisposition for HTA are associated with liver-related outcomes in the population. New initiation of antihypertensive medication attenuates this association in persons with high genetic risk for HTA.

## Introduction

Arterial hypertension (HTA) is one of the most important risk factors for cardiovascular morbidity and mortality^1^. Observational studies, dating back to the 1970s, also link HTA with chronic liver disease, especially non-alcoholic fatty liver disease (NAFLD)^2–4^. NAFLD is currently the most common chronic liver disease affecting around 25% of the adult population^5^.

Although NAFLD and HTA share many metabolic features, the relationship between NAFLD and HTA seems to be independent of these other metabolic components^4^. Specifically, HTA has been associated with elevated liver enzymes, liver steatosis and advanced fibrosis, independent of body mass index (BMI), metabolic factors (e.g. abdominal obesity, diabetes, dyslipidemia), alcohol use and other relevant confounders in numerous studies^3,6–18^. For example, a cross-sectional study based on data from 16,082 individuals in 26 cohorts found systolic blood pressure (SBP) to be independently associated with advanced liver fibrosis^17^. Furthermore, a systematic review of studies on NAFLD with paired liver biopsies identified HTA as a predictor of more rapid liver fibrosis progression over time, whereas metabolic syndrome, diabetes or BMI failed to predict such progression^19^. Recently, large population studies found HTA to be associated with severe liver-related outcomes including hepatocellular carcinoma independently of relevant confounders^20–24^. HTA was even reported to have a stronger risk effect for future liver cirrhosis than other metabolic traits such as diabetes, dyslipidemia, and obesity^21^.

Despite these previous observations, it still remains unclear whether the association between HTA and liver disease is causal or due to unmeasured or inadequately measured confounding secondary to, for example, alcohol use, other lifestyle factors, or associated metabolic conditions. Assuming that a causal relationship between HTA and liver disease exists, it remains unclear whether this relationship is modifiable by antihypertensive medication.

Many lifestyle factors may affect blood pressure over time, and therefore genetic predisposition to HTA may serve as a better marker than measured blood pressure to study associations with other conditions, such as liver disease. Polygenic risk scores (PRS) provide an overall measure of an individual’s genetic predisposition to develop a condition such as HTA^25,26^.

The aim of this study was to analyze whether HTA is associated with liver-related outcomes. For this purpose, we analyzed both clinically defined HTA and a recently published hypertension-PRS in a large general population cohort with linked electronic healthcare registry data for liver-related outcomes. By linkage with national drug imbursement registries, we further investigated whether use of antihypertensive medication influences the association between the genetic predisposition for HTA and liver disease.

## Methods

Data were from the Finnish health-examination surveys, FINRISK 1992, 1997, 2002, 2007, and 2012, as well as the Health 2000 Survey. The FINRISK studies are cross-sectional health-examination surveys conducted in Finland every five years in a systematic and standardized fashion since 1972 by the Finnish Institute for Health and Welfare (previously National Public Health Institute). The surveys provide data on representative samples of adults (25–74 years) from 4 to 6 regions in Finland. The samples were randomly drawn from the Finnish National Population Register and were stratified by region, gender, and 10-year age groups^27^.

The Health 2000 Survey, conducted in 2000–2001, was also coordinated by the Finnish Institute for Health and Welfare (previously National Public Health Institute), and the cohort is considered representative of the adult Finnish population through a regional two-stage stratified cluster sampling procedure^28^.

Data were collected at baseline via interviews, questionnaire, health examination, and blood sampling as previously described^27,28^. All participants provided signed informed consent, and the studies were approved by the Coordinating Ethical Committee of the Helsinki and Uusimaa Hospital District (previously studies also approved by the institutional review board of the National Public Health Institute, both in Helsinki, Finland). The FINRISK and Health 2000 sample collections were transferred to THL Biobank in 2015 after approval of the Coordinating Ethical Committee of the Helsinki and Uusimaa Hospital District. All methods were performed in accordance with the relevant guidelines and regulations and with the Declaration of Helsinki.

SBP and diastolic blood pressure (DBP) were measured two or three times from the right arm in the sitting position using the mercury sphygmomanometer following at least 5 min of rest; we used the mean value of these measurements in this study^27,28^. Respondents were asked to report how often they consumed alcoholic beverages during the previous year and the average amount they consumed per week during the previous month. Average alcohol intake (100% ethanol in grams per day) was calculated based on these data. Smoking status was categorized as never smokers, previous smokers and current smokers. Exercise was assessed by asking how often the subject performs leisure-time physical exercise for at least 20–30 min so that he/she is at least slightly out of breath and sweaty. Diabetes was defined either by a fasting serum glucose ≥ 7.0 mmol/L (126 mg/dL), taking diabetic medication, or by having a prior known diabetes diagnosis. BMI (kg/m^2^) and waist circumference (cm) were measured at baseline^27,28^.

The FINRISK and Health 2000 samples were genotyped using Illumina CoreExome, OMNIExpress, and 610 K arrays. Individuals with non-Finnish genetic ancestry, as defined using principal component analysis, or obscure sex were excluded. Quality control before phasing and imputation excluded variants with missingness > 5%, call rate < 95%, minor allele count (MAC) < 3 (if Zcalled) or MAC < 10 (if called using Illumina GenCal), INFO < 0.8, minor allele frequency < 0.001%, Hardy–Weinberg equilibrium *p*-value < 1 * 10–10, and heterozygosity exceeding ± 4 standard deviations. The quality control was performed simultaneously on all data. Prior to imputation, the haplotypes were estimated using Eagle 2.3.5^29^. Imputation was performed with Beagle 4.1^30^ using high-coverage, population-specific SISu v3 reference panel of 3775 whole-genome sequences at the Institute for Molecular Medicine Finland (FIMM).

PRSs for SBP and DBP were derived from GWAS where over 1 million people were studied^26^. To generate PRSs we used GWAS summary statistics from discovery meta-analysis comprising 757,601 individuals from the UK Biobank and International Cancer Benchmarking Partnership (ICBP) samples that were publicly available^31^. PRS was constructed by calculating the weighted sum of risk alleles which an individual carry. PRS weights were calculated with PRS-CS^32,33^ using European 1000 Genomes Project samples as an external LD reference panel. Polygenic score for each individual was produced using PLINK’s –score command^34^. The mean of the PRS was scaled to zero with R 3.6.0 to reflect SD scaling in addition to mean centering. The final variant counts of the PRSs were 1,072,098 for SBP and 1,073,588 for DBP.

Follow-up data were obtained from several national electronic healthcare registers through linkage using the unique personal identity code assigned to all Finnish residents. The Finnish Drug Reimbursement Register covers all reimbursed pharmacy prescription drug purchases since 1964, purchase timestamps, and respective Anatomical Therapeutic Chemical Classification System codes (ATC). In the present study, exposure to antihypertensive medication required at least three purchase events for drugs with ATC codes C02, C03, C07, C08, and C09, and start of exposure was the time-point of the first purchase event. All of these drugs are reimbursed prescription drugs in Finland and therefore registry coverage is complete. Data for hospitalizations were obtained from the Care Register for Health Care (HILMO), which covers all hospitalizations in Finland since 1969. One or several ICD-diagnoses are assigned to each hospitalization at discharge; these diagnosis codes are systematically recorded in the HILMO register. Data for malignancies were obtained from the Finnish Cancer Registry, with nationwide cancer records since 1953. Vital status and cause of death data were obtained from Statistics Finland. In Finland, each person who dies is by law assigned a cause of death (in accordance with the ICD) to the official death certificate, issued by the treating physician based on medical or autopsy evidence, or forensic evidence when necessary; the death codes are then verified by medical experts at the register and recorded according to systematic coding principle. Data collection to all these registries is mandatory by law and general quality is consistent and virtually 100% complete^35,36^.

Study endpoints were fatal and non-fatal liver disease requiring hospital admission or causing liver cancer or liver-related death, and defined by the ICD-10 codes K70-K77 or C22.0, or ICD-8/9 codes 570–573 or 155.0 in any of the follow-up registries. Regarding hospitalizations, we only considered liver events recorded as either the main cause of hospitalization or as the first or second side cause.

From the combined FINRISK and Health 2000 dataset (n = 43,105), we excluded subjects with missing registry-follow-up (n = 1506), missing genetic data (n = 5221), chronic viral hepatitis (n = 100), one individual from each related pair with a kinship coefficient > 0.2 or duplicates (n = 2270), or a record of liver disease at or before baseline (n = 238). The remaining cohort comprised 33,770 subjects.

### Statistical analyses

For comparing groups, we used Chi Square, Student’s t-test or Mann Whitney tests as appropriate. Associations between baseline variables and SBP PRS and DBP PRS was analyzed by linear regression adjusted for age and sex. Cox regression analyses were performed with time to the first liver event as the outcome. The following HTA-related exposure variables were analyzed in separate Cox models: measured SBP, measured DBP, SBP PRS, DBP PRS, elevated blood pressure (systolic blood pressure ≥ 130 or diastolic ≥ 85 mmHg or antihypertensive medication use as defined by Alberti et al.^37^), and HTA status. HTA status was defined in line with recent guidelines^38^ by a blood pressure cutoff of ≥ 140 (systolic) or ≥ 90 (diastolic) mmHg and baseline antihypertensive medication use in the following categories: (a) normal blood pressure, no medication; (b) high blood pressure, previously unknown; (c) high blood pressure, previously known, no medication; (d) high blood pressure despite medication; and (e) normal blood pressure during medication. Two sets of adjustments were considered: adjustment for (a) age and sex, or adjustment for (b) age, sex, BMI, waist circumference, weekly alcohol use, fraction of alcohol use as wine, low-density lipoprotein (LDL) cholesterol, high-density lipoprotein (HDL) cholesterol, triglycerides, diabetes, exercise habits, smoking status and baseline cardiovascular disease. In addition to this, Cox regression analyses with genetic risk scores as covariates were also adjusted for genotyping chip, and the first three principal components of genetic structure. Two-way interactions for the liver outcome were tested between the HTA PRSs and alcohol use, BMI, waist circumference, diabetes, and prevalent cardiovascular disease. A subgroup analysis was performed by the presence of the metabolic syndrome at baseline. Metabolic syndrome was defined according to the Joint Interim Statement criteria^37^, using the following cutoffs: waist circumference ≥ 94 cm for men and ≥ 80 cm for women; serum triglycerides ≥ 1.7 mmol/L; serum HDL-cholesterol < 1.0 mmol/L for men and < 1.3 mmol/L for women; and fasting serum glucose ≥ 5.6 mmol/L or diabetes medication.

To investigate whether antihypertensive medication modifies the association between HTA and liver-related outcomes, we analyzed interaction effects between antihypertensive medication use and SBP PRS and DBP PRS by Cox regression with confounder adjustments as above. To avoid prevalent-user bias, we excluded subjects with antihypertensive medication use at or before baseline from these analyses. To mitigate immortal-time bias, medication exposure was modelled as a time-varying variable using the tmerge function of the R survival package. We also performed a sensitivity analysis excluding subjects with antihypertensive medication initiated > 5 years after baseline. The functional form of the SBP PRS and DBP PRS in interaction with antihypertensive medication was plotted using restricted cubic splines. For each Cox regression model and for each covariate used, we evaluated the proportional hazards assumption both visually by plotting Schoenfeld residuals against time and by testing with the score test whether the slope of the corresponding time-dependent coefficient deviates from zero (R’s cox.zph-function). Data were analyzed with R software version 3.6.1^39^.

### Ethics approval

The studies were approved by the Coordinating Ethical Committee of the Helsinki and Uusimaa Hospital District (previously studies also approved by the institutional review board of the National Public Health Institute, both in Helsinki, Finland). The FINRISK and Health 2000 sample collections were transferred to THL Biobank in 2015 after approval of the Coordinating Ethical Committee of the Helsinki and Uusimaa Hospital District.

### Patient consent

All participants provided signed informed consent.

## Results

The study comprised 33,770 individuals with a mean age of 49.6 years and 54% women (Table 1). Mean SBP and DBP were 135 (SD 20) and 81 (SD 11) mmHg, respectively. Seventy-two percent had elevated blood pressure (blood pressure ≥ 130/ ≥ 85 mmHg or antihypertensive medication). Measured SBP and DBP correlated significantly with the respective SBP and DBP PRSs (Supplementary Fig. 1). Of baseline variables, age, sex, measured blood pressures, diabetes, metabolic syndrome, cardiovascular disease, alcohol intake, fraction of alcohol intake as wine, exercise, and triglycerides were associated with both SBP and DBP PRSs when assessed using age- and sex-adjusted linear regression analysis (Table 1 and Supplementary Table 1).

**Table 1:** Baseline demographics.

	All subjects	Medication-naïve non-initiator	Medication naïve new-initiator	*P*	*P* for association with SBP PRS^b^	*P* for association with DBP PRS^b^
Persons	33,770	16,150	9576			
Age	49.6 (13.9)	43.8 (12.5)	50.4 (12.2)	< 0.001	0.008	0.011
Women	18,075 (53.5)	8643 (53.5)	5094 (53.2)	0.626	0.022	0.025
SBP	134.6 (19.8)	126.7 (15.8)	140.6 (19.8)	< 0.001	< 0.001	< 0.001
DBP	80.7 (11.4)	77.3 (10.3)	84.5 (11.3)	< 0.001	< 0.001	< 0.001
Pulse pressure	54.0 (15.8)	49.5 (12.8)	56.2 (16.2)	< 0.001	< 0.001	< 0.001
Elevated blood pressure^a^	24,140 (71.5)	7029 (43.6)	9289 (97.0)	< 0.001	< 0.001	< 0.001
Body mass index	26.8 (4.7)	25.5 (4.1)	27.3 (4.7)	< 0.001	0.152	0.023
Waist circumference	90.3 (13.8)	86.5 (12.4)	91.0 (13.7)	< 0.001	0.492	0.339
Diabetes	2589 (7.7)	470 (2.9)	586 (6.1)	< 0.001	< 0.001	< 0.001
Metabolic syndrome	11,135 (33.0)	2758 (17.1)	3910 (40.8)	< 0.001	< 0.001	< 0.001
Cardiovascular disease	1229 (3.6)	77 (0.5)	148 (1.5)	< 0.001	< 0.001	< 0.001
Weekly alcohol use (grams/week)	75.0 (137.4)	77.9 (134.6)	75.8 (136.5)	0.237	0.003	0.024
Fraction of alcohol use as wine	0.23 (0.34)	0.24 (0.34)	0.22 (0.34)	0.010	< 0.001	0.002
**Smoking status**				< 0.001		
Current	8013 (23.9)	4241 (26.4)	2447 (25.7)		0.395	0.262
Former	7590 (22.6)	3147 (19.6)	2171 (22.8)		0.216	0.062
Never	17,920 (53.5)	8691 (54.1)	4900 (51.5)		Ref	Ref
**Exercise**				< 0.001		
At least 2 times a week	16,137 (57.3)	7561 (58.4)	5010 (54.4)		Ref	Ref
2–4 times a month	7526 (26.7)	3642 (28.1)	2703 (29.3)		< 0.001	0.002
Less often	4481 (15.9)	1753 (13.5)	1505 (16.3)		0.078	0.647
Low-density lipoprotein cholesterol	3.41 (0.97)	3.34 (0.95)	3.59 (0.97)	< 0.001	0.497	0.659
High-density lipoprotein cholesterol	1.43 (0.38)	1.46 (0.38)	1.41 (0.38)	< 0.001	0.001	0.108
Triglycerides	1.47 (1.00)	1.33 (0.89)	1.56 (1.06)	< 0.001	< 0.001	< 0.001

Data are as mean (SD) or n (%).

HTA, arterial hypertension; SBP, systolic blood pressure; DBP, diastolic blood pressure; PRS, polygenic risk score; Ref, reference group.

^a^Either measured blood pressure ≥ 130 (systolic) or ≥ 85 (diastolic) mmHg or antihypertensive medication use at baseline.

^b^By age- and sex-adjusted linear regression analysis.

During a median follow-up of 12.9 years (IQR 7.8–17.8, range 0.1–23.0, 409,268.9 person-years), we observed 441 liver-related outcomes (262 among men and 179 among women) and 3642 deaths (2147 among men and 1495 among women).

**Figure 1:**
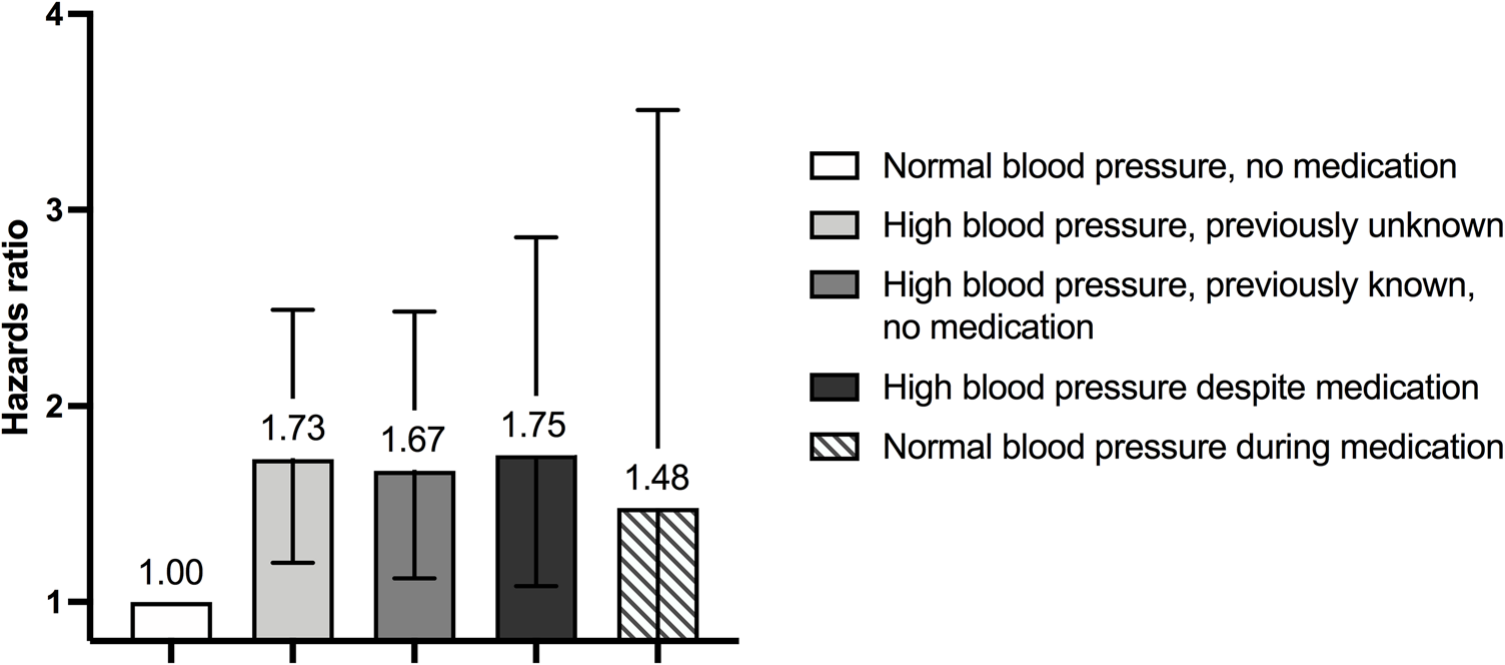
Associations by Cox regression between different categories of baseline arterial hypertension and liver-related outcomes. High blood pressure is defined as measured blood pressure ≥ 140 (systolic) or ≥ 90 (diastolic) mmHg. Error bars reflect 95% confidence intervals.

In separate Cox regression models adjusted for age and sex, measured SBP, DBP and pulse pressure were associated with incident liver-related outcomes (Table 2). In the fully adjusted models, SBP and pulse pressure remained significantly associated with liver-related outcomes. Elevated blood pressure as a categorical variable (blood pressure ≥ 130/85 mHg or antihypertensive medication) exhibited a hazards ratio (HR) of 1.8 in the fully adjusted model (Table 2). When considering the multicategorical HTA status (categories = (a) normal blood pressure, no medication; (b) high blood pressure, previously unknown; (c) high blood pressure, previously known, no medication; (d) high blood pressure despite medication; and (e) normal blood pressure during medication, with high blood pressure defined as ≥ 140 (systolic) or ≥ 90 (diastolic) mmHg) as the independent variable in the fully adjusted model, all categories depicting HTA showed increased rates of liver-related outcomes compared to those with normal blood pressure without medication (Table 2 and Fig. 1). The highest rates of liver outcomes were observed among those with high blood pressure despite medication and those with previously unknown HTA, but the confidence intervals were overlapping between categories.

**Table 2:** Associations between hypertension and liver-related outcomes using both measured blood pressure, use of antihypertensive medication and polygenic risk scores (PRSs).

	n/N	HR^a^ (95% CI)	*P*	HR^b^ (95% CI)	*P*
Measured SBP (per 1 SD)	441/33,770	1.22 (1.10–1.34)	< 0.001	1.16 (1.03–1.30)	0.01
Measured DBP (per 1 SD)	441/33,770	1.17 (1.07–1.29)	0.001	1.06 (0.95–1.20)	0.29
Measured pulse pressure (per 1 SD)	441/33,770	1.12 (1.02–1.23)	0.019	1.12 (1.00–1.24)	0.04
**Elevated blood pressure**^c^					
No	61/9602	1.00		1.00	
Yes	380/24,140	1.92 (1.44–2.54)	< 0.001	1.79 (1.27–2.51)	0.001
**HTA status**^d^					
Normal blood pressure, no medication	104/11,361	1.00		1.00	
High blood pressure, previously unknown	72/4444	1.47 (1.07–2.01)	0.02	1.73 (1.20–2.49)	0.003
High blood pressure, previously known, no medication	70/3304	2.07 (1.51–2.83)	< 0.001	1.67 (1.12–2.48)	0.01
High blood pressure despite medication	40/2478	1.86 (1.25–2.76)	0.002	1.75 (1.08–2.86)	0.02
Normal blood pressure during medication	9/870	1.57 (0.78–3.14)	0.21	1.48 (0.63–3.51)	0.37
SBP PRS (per 1 SD)	441/33,770	1.12 (1.02–1.23)	0.02	1.19 (1.01–1.24)	0.04
DBP PRS (per 1 SD)	441/33,770	1.13 (1.03–1.24)	0.01	1.12 (1.01–1.25)	0.03

Analyses are by Cox regression analyses.

CI, confidence interval; HR, hazards ratio; HTA, arterial hypertension; SBP, systolic blood pressure; DBP, diastolic blood pressure; PRS, polygenic risk score.

^a^Adjusted for age and sex. Analyses including polygenic risk scores (PRS) are additionally adjusted for genotyping chip and the first three principal components of genetic structure.

^b^Adjusted for age, sex, body mass index, waist circumference, weekly alcohol use, fraction of alcohol use as wine, low-density lipoprotein cholesterol, high-density lipoprotein cholesterol, triglycerides, diabetes, exercise habits, smoking status (current, former, never smoker) and baseline cardiovascular disease. Analyses including polygenic risk scores (PRS) are additionally adjusted for genotyping chip and the first three principal components of genetic structure.

^c^Either measured blood pressure ≥ 130 (systolic) or ≥ 85 (diastolic) mmHg or antihypertensive medication use at baseline.

^d^High blood pressure defined as ≥ 140 (systolic) or ≥ 90 (diastolic) mmHg.

In separate Cox regression models considering the PRSs as independent variables, both the SBP and DBP PRSs were significantly associated with liver-related outcomes in both age- and sex-adjusted models and in the fully adjusted models (Table 2). Interaction terms between SBP PRS or DBP PRS and alcohol use, BMI, waist circumference, diabetes, and prevalent cardiovascular disease were all non-significant (Supplementary Table 2).

We next studied possible effect modification from the metabolic syndrome. In Cox regression analysis with age, sex, alcohol use, SBP or DBP PRS, metabolic syndrome, and the interaction terms between the PRSs and metabolic syndrome as covariates, the risk for liver-related outcomes increased along with the SBP PRS and DBP PRS both among those with and without the metabolic syndrome (Supplementary Fig. 2). Both SBP PRS and DBP PRS were significantly associated with liver-related outcomes among subjects without baseline metabolic syndrome (SBP PRS: HR 1.19, 95% CI 1.00–1.42, *P* = 0.049; DBP PRS: HR 1.28, 95% CI 1.07–1.53, *P* = 0.006, adjusted for age, sex, and alcohol use).

### Antihypertensive medication modifies the association between genetic predisposition for HTA and liver-related outcomes

After excluding 8044 individuals with antihypertensive medication use at or before baseline, the cohort comprised 16,150 medication-naïve non-initiators and 9576 medication-naïve new initiators (Table 1). Among the new initiators, mean time from study baseline to initiation of medication was 7.4 years (median 6.3 years, IQR 3.1–10.6). During follow-up, there were 180 liver-related outcomes among non-initiators and 141 among new initiators.

**Figure 2:**
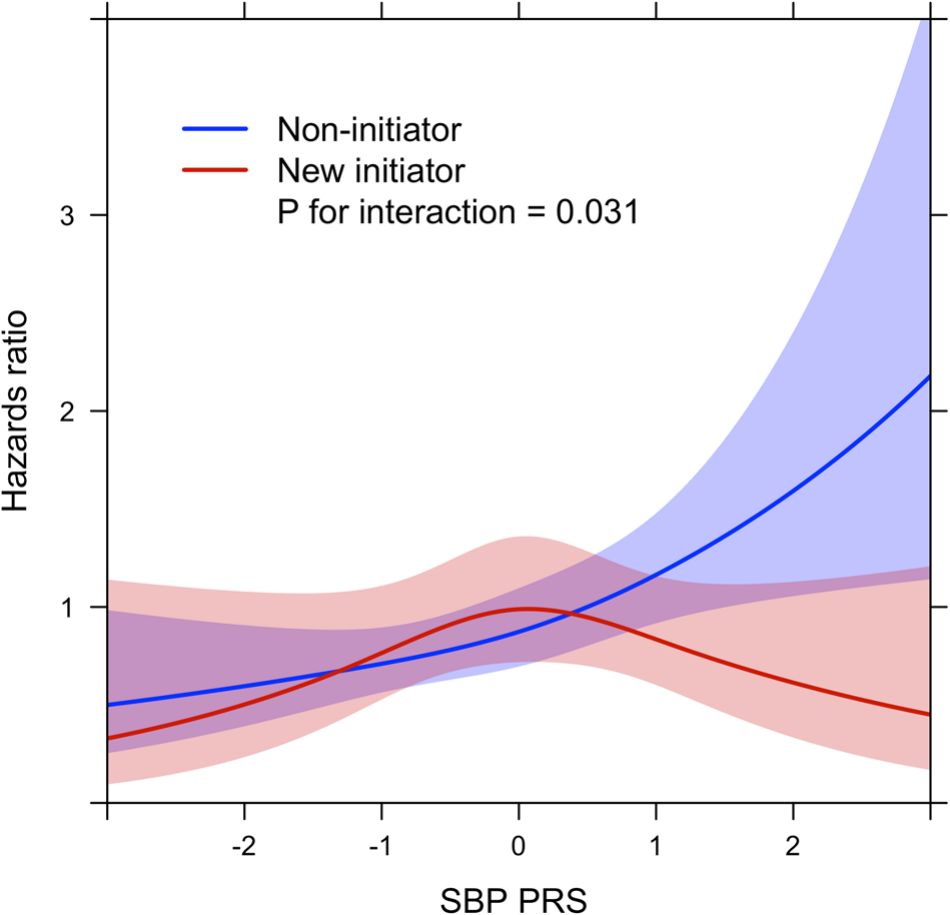
Interaction between systolic blood pressure polygenic risk score (SBP PRS) and new initiation of antihypertensive medication for liver-related outcomes by time-dependent Cox regression analysis using restricted cubic splines. SBP PRS is standardized as per 1 SD. The light red and light blue areas reflect 95% confidence intervals.

**Figure 3:**
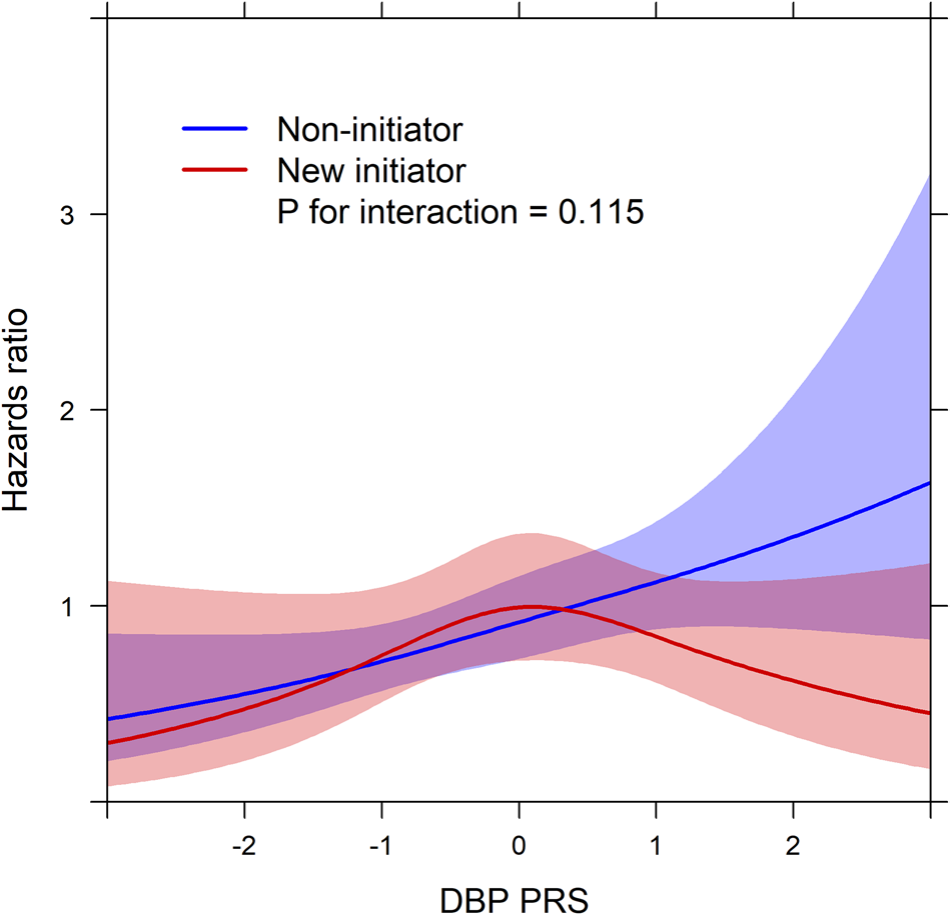
Interaction between diastolic blood pressure polygenic risk score (DBP PRS) and new initiation of antihypertensive medication for liver-related outcomes by time-dependent Cox regression analysis using restricted cubic splines. DBP PRS is standardized as per 1 SD. The light red and light blue areas reflect 95% confidence intervals.

SBP PRS and DBP PRS were significantly associated with liver-related outcomes independently of antihypertensive medication exposure in the fully adjusted models (Table 3). Initiation of medication after baseline was non-significant. However, when accounting for non-linearity of the PRSs, there was a tendency towards reduced rates of liver outcomes among medication new initiators at the highest end of the SBP and DBP PRSs (Figs. 2 and 3). In fact, in the highest quintile of the SBP PRS (n = 5058 with 80 liver-related outcomes), new initiation of antihypertensive medication was associated with a reduced rate of liver-related outcomes (HR 0.55, 95% CI 0.31–0.97, *P* = 0.039, fully adjusted Cox model).

**Table 3:** Joint associations and interaction between hypertension polygenic risk scores (PRSs) and antihypertensive medication for liver-related outcomes among 25,726 subjects that were medication-naïve at baseline. Analyses are by Cox regression analyses.

	Overall (n = 25,726)		Excluding subjects with medication started > 5 years after baseline (n = 23,855)	
	HR^a^ (95% CI)	*P*	HR^a^ (95% CI)	*P*
**Model with SBP PRS and medication**				
SBP PRS (per 1 SD)	1.28 (1.13–1.46)	< 0.001	1.33 (1.14–1.55)	< 0.001
Antihypertensive medication	0.93 (0.72–1.21)	0.598	0.76 (0.53–1.08)	0.127
Interaction term SBP PRS * antihypertensive medication	0.79 (0.63–0.98)	0.038	0.73 (0.53–1.02)	0.064
**Model with DBP PRS and medication**				
DBP PRS (per 1 SD)	1.25 (1.10–1.42)	< 0.001	1.37 (1.18–1.59)	< 0.001
Antihypertensive medication	0.94 (0.72–1.21)	0.615	0.71 (0.49–1.03)	0.074
Interaction term DBP PRS * antihypertensive medication	0.81 (0.65–1.02)	0.072	0.93 (0.67–1.29)	0.661

CI, confidence interval; HR, hazards ratio; SBP, systolic blood pressure; DBP, diastolic blood pressure; PRS, polygenic risk score.

^a^Adjusted for age, sex, body mass index, waist circumference, weekly alcohol use, fraction of alcohol use as wine, low-density lipoprotein cholesterol, high-density lipoprotein cholesterol, triglycerides, diabetes, exercise habits, smoking status (current, former, never smoker) and baseline cardiovascular disease, genotyping chip and the first three principal components of genetic structure.

In a sensitivity analysis excluding 1871 subjects with antihypertensive medication initiated > 5 years after baseline, the findings were fairly similar to those in the overall population (Table 3).

## Discussion

We found that genetic predisposition to HTA is independently associated with incident liver-related outcomes in the general population. A similar association was found for HTA defined by blood pressure measurements and antihypertensive medication use at baseline. Furthermore, initiation of antihypertensive medication after study baseline was associated with a reduction in the rate of liver-related outcomes in individuals with a high genetic risk of HTA compared to a lack of antihypertensive medication exposure in such individuals.

Strengths of our study include the large and well-characterized health-examination survey cohort considered representative of the general population, with linkage to comprehensive and reliable national healthcare registers^27,28^. Data regarding antihypertensive medication use from the drug reimbursement register are also virtually complete. We were able to account for medications started any time during the follow-up, which allowed modelling drug exposure in a time-dependent fashion. Genetic risk of hypertension, as assessed by the previously developed PRSs^26^, overcomes issues with the time-varying nature of measured blood pressures and hypertension as exposures, and is subject to less confounding than single blood pressure measurements. Furthermore, measured blood pressure may be a confounder on the causal pathway between alcohol and liver disease; specifically, alcohol intake may be underestimated and/or high blood pressure might reflect increased sensitivity to alcoholic toxicity. The PRSs should not be affected by such issues.

Study limitations include the inability to account for time-varying confounding since phenotype variables were recorded only at study baseline. Still, the PRSs are unaffected by changes in lifestyle and environmental factors over time. Regarding antihypertensive medication, initiation of medication late after study baseline probably means that the person has over time acquired additional risk factors of HTA, many of which are also risk factors for liver disease. In this situation, adjustment for time-varying confounding would expectedly amplify the beneficial impact on liver-related outcomes of antihypertensive medication in the high-risk PRS groups, but this remains to be shown.

Baseline alcohol use was defined by questionnaires with its inherent limitations. Also, data on medication type and doses used were unavailable, and the statistical power to address this was considered inadequate. The drug exposure analysis represents intention to treat, and duration of treatment was not explicitly defined. Antihypertensive medications can also be used for other indications than blood pressure treatment; nonetheless, this should not affect the interaction effects between antihypertensive medication and genetic HTA risk for liver outcomes.

Numerous previous studies have reported associations between HTA and liver enzymes, steatosis, fibrosis, and liver-related outcomes^3,6–24^. One study even found that the association with liver-related outcomes was stronger for HTA than for other metabolic traits^21^. None of these previous studies, however, used polygenic risk scores to define hypertension.

Although our findings together with previous ones support the existence of a link between HTA and liver disease, possible pleiotropy and residual confounding may still prevent definitive conclusions. For the same reasons, the assumptions of Mendelian randomization analysis do not hold^40^, and we did therefore not engage in Mendelian randomization analyses in this study. Future studies with larger sample sizes should investigate specific genetic variants to elucidate possible mechanisms between HTA and liver disease.

Proposed mechanisms linking HTA with liver disease include activation of the renin–angiotensin–aldosterone system (RAAS), insulin resistance, and endothelial dysfunction-related arterial stiffness^4^. Experimental studies suggest a role of the RAAS in the progression of liver fibrosis^41–45^, and RAAS inhibitors have been associated with beneficial effects on the liver in a multitude of animal studies^46–48^. It has also been shown that activated human hepatic stellate cells, which are responsible for fibrogenesis in the liver, express components of the RAAS and synthesize angiotensin II, suggesting that locally generated angiotensin II could participate in tissue remodeling in the human liver^43^. A trend towards improvements in liver enzyme levels with the use of RAAS inhibitors has been found in clinical trials of NAFLD patients, but results are inconsistent and studies have generally been small with short duration of follow-up^49^. One observational study with a median follow-up of 36 months reported that the use of RAAS inhibitors in patients with NAFLD and type 2 diabetes was associated with a slower liver fibrosis progression rate^50^. Prospective trials with long follow-up are needed to confirm whether a good blood pressure control can contribute to slowing down the progression of liver fibrosis and decreasing the risk for liver-related clinical outcomes, and whether such potential effects are related to specific medication types (e.g. RAAS inhibitors) or antihypertensive treatment in general.

In conclusion, HTA was independently associated with liver disease. Such an association was found both for HTA defined clinically and by polygenic risk scores. New initiation of antihypertensive medication was associated with reduced rates of liver-related outcomes in persons with high genetic HTA risk.

## Supplementary Information

Supplementary Information.

## Data Availability

FINRISK and Health 2000 data are available from the THL biobank based on a research application, as explained on the website of the THL biobank (https://thl.fi/en/web/thl-biobank/for-researchers).

## Abbreviations

HTA: Arterial hypertension
PRS: Polygenic risk score
NAFLD: Non-alcoholic fatty liver disease
BMI: Body mass index
SBP: Systolic blood pressure
DBP: Diastolic blood pressure
MAC: Minor allele count
ATC: Anatomical Therapeutic Chemical Classification System codes
HILMO: Care Register for Health Care
LDL: Low-density lipoprotein
HDL: High-density lipoprotein
HR: Hazards ratio
CI: Confidence interval
