# Supplementary material for: Association between arterial hypertension and liver outcomes using polygenic risk scores: a population-based study
Source: Sci Rep. 2022 Sep 16;12:15581. doi: 10.1038/s41598-022-20084-z (PMC9481629; doi:10.1038/s41598-022-20084-z)
Supplement: Supplementary file 1 — Supplementary Information. [file 41598_2022_20084_MOESM1_ESM.docx]

**SUPPLEMENTARY MATERIAL**

Supplementary figure 1. Association between measured blood pressure and polygenic risk scores by linear regression analysis with restricted cubic splines to account for non-linear association, for (A) systolic blood pressure and (B) diastolic blood pressure. The gray areas reflect 95% confidence intervals.

(A)


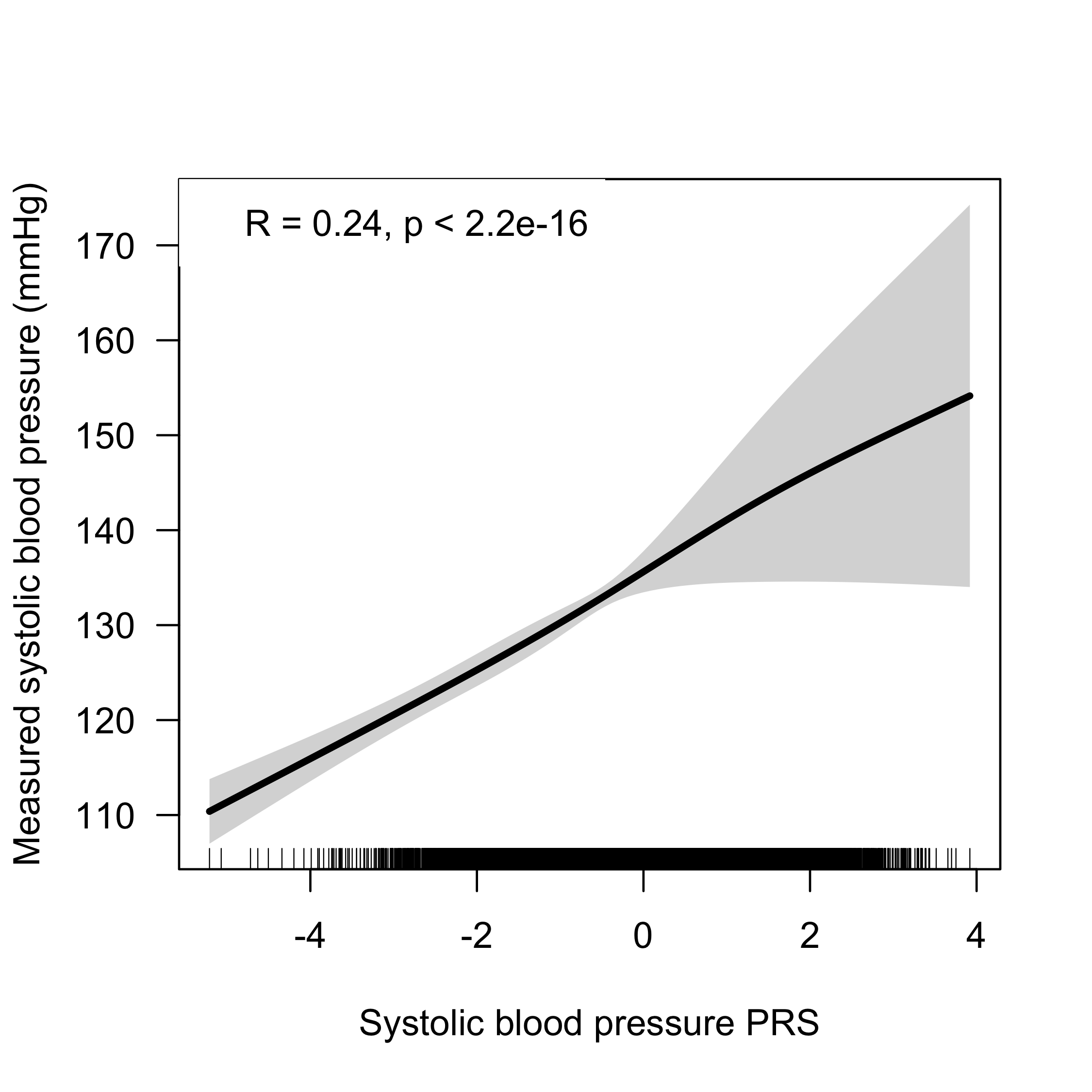


(B)


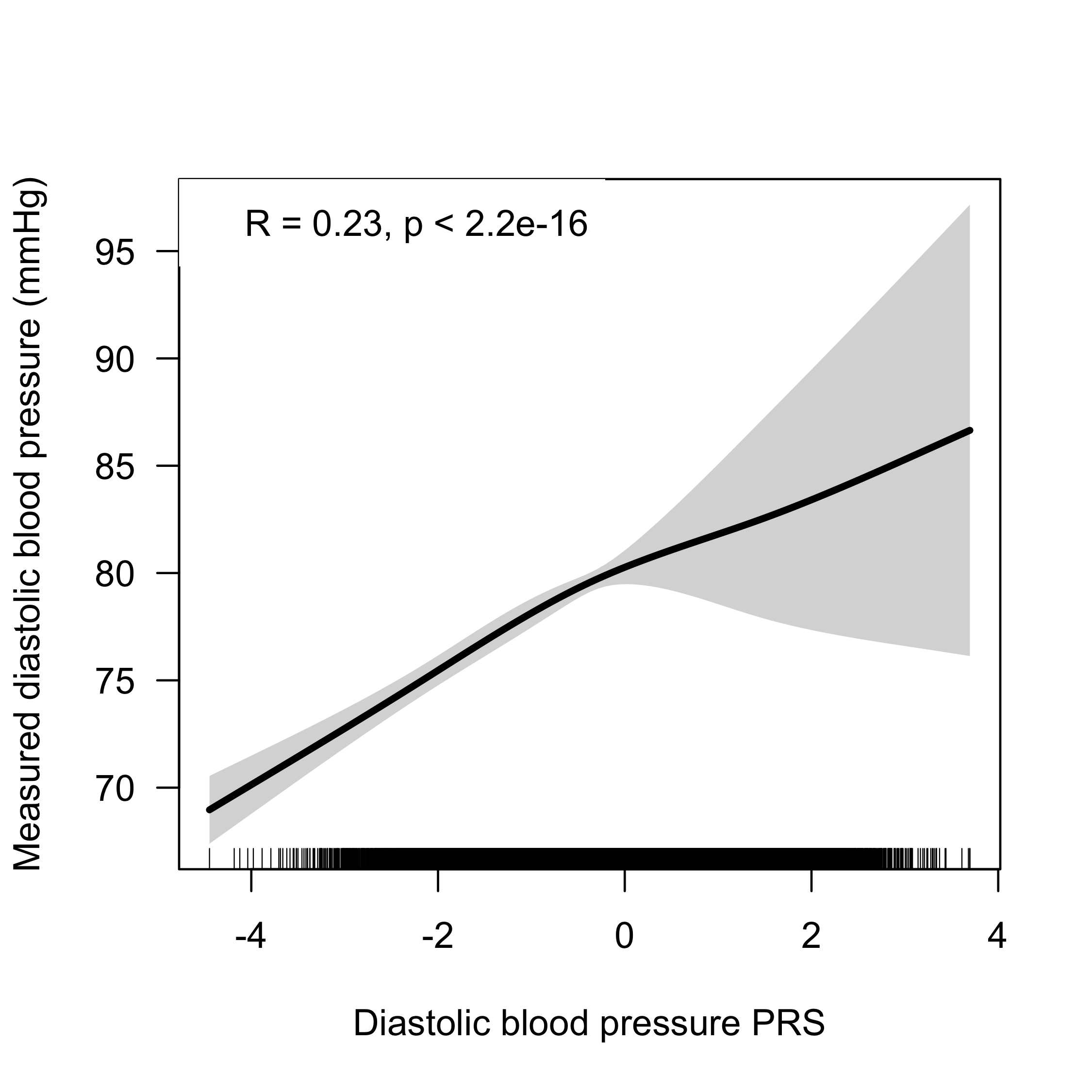


Supplementary figure 2. Association between (A) the systolic blood pressure polygenic risk score (SBP PRS) and (B) the diastolic blood pressure PRS (DBP PRS) with liver-related outcome stratified by the presence of baseline metabolic syndrome (MetS). Analyses are by Cox regression adjusted for age, sex, and alcohol use, and including the interaction term between metabolic syndrome and SBP PRS or DBP PRS. The light red and light blue areas reflect 95% confidence intervals.

(A)


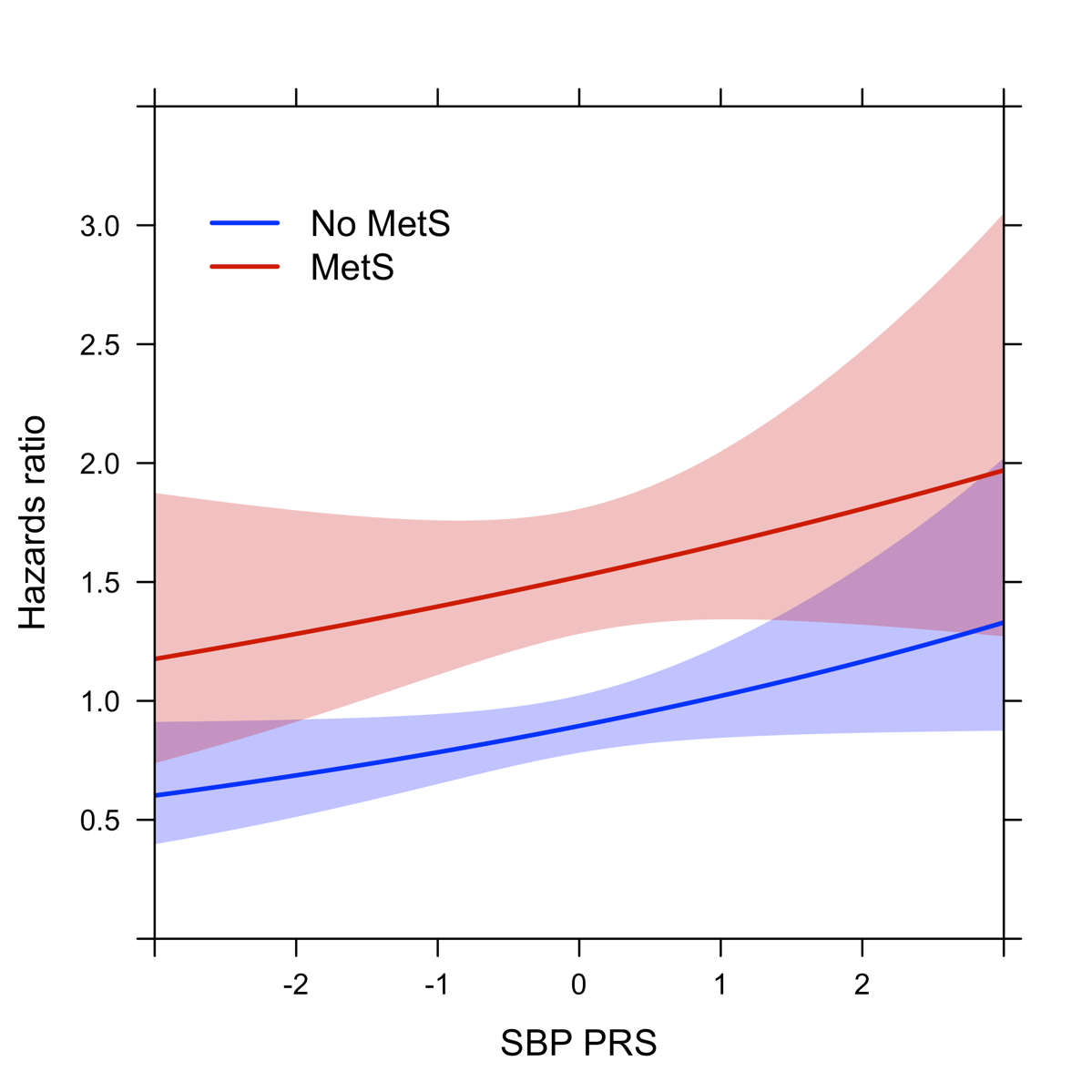


(B)


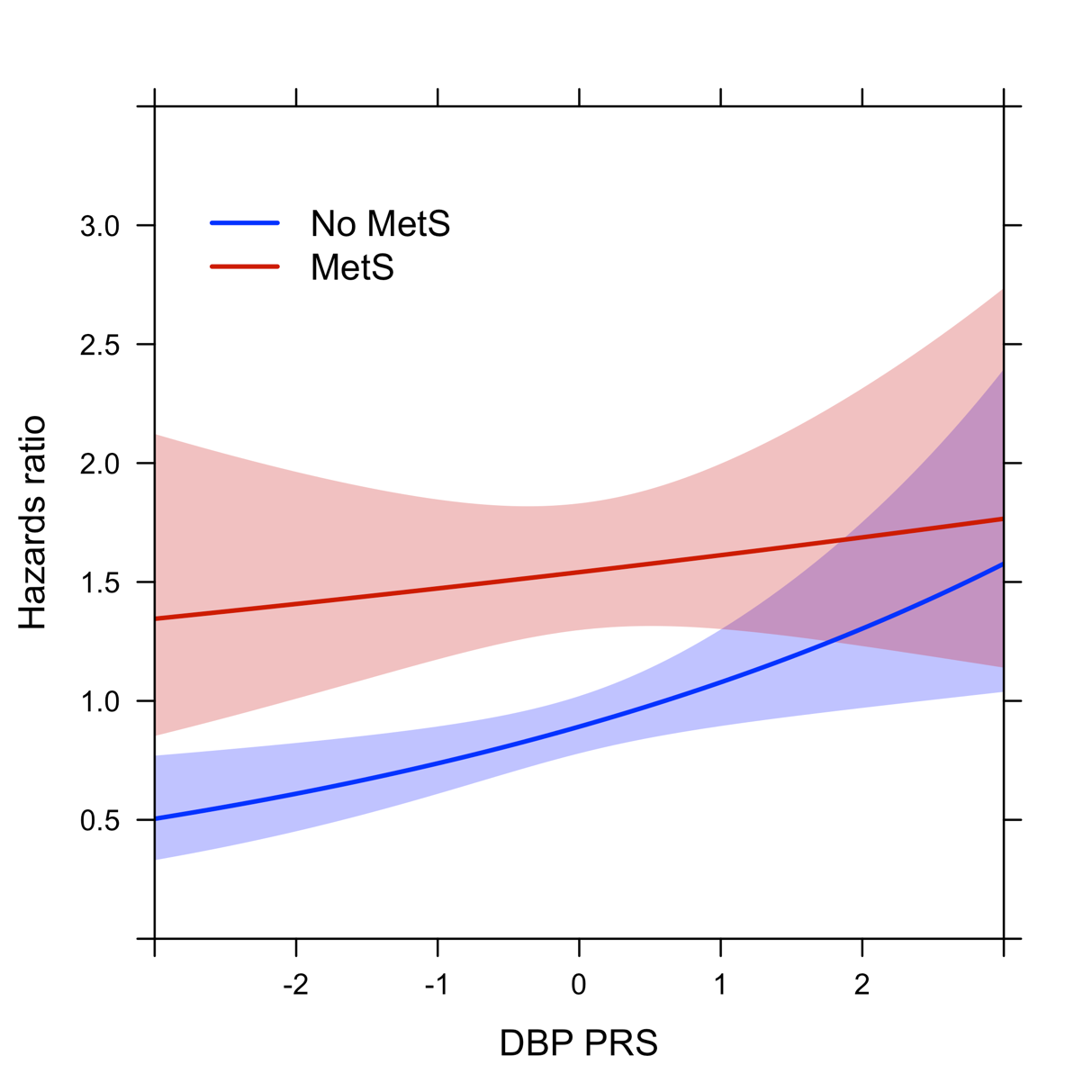


Supplementary table 1. The association between baseline variables and systolic and diastolic blood pressure polygenic risk scores (PRS) by age- and sex-adjusted linear regression analysis.

|  | **Systolic blood pressure PRS** | | | **Diastolic blood pressure PRS** | | |
| --- | --- | --- | --- | --- | --- | --- |
|  | Coefficient | Standard error | P-value | Coefficient | Standard error | P-value |
| Age | -0.001 | 0.000 | 0.008 | -0.001 | 0.000 | 0.011 |
| Women | 0.025 | 0.011 | 0.022 | 0.025 | 0.011 | 0.025 |
| SBP | 0.016 | 0.000 | <0.001 | 0.013 | 0.000 | <0.001 |
| DBP | 0.017 | 0.000 | <0.001 | 0.023 | 0.001 | <0.001 |
| Pulse pressure | 0.014 | 0.000 | <0.001 | 0.006 | 0.000 | <0.001 |
| Elevated blood pressure^1^ | 0.517 | 0.013 | <0.001 | 0.501 | 0.013 | <0.001 |
| Body mass index | -0.002 | 0.001 | 0.152 | -0.003 | 0.001 | 0.023 |
| Waist circumference | -0.003 | 0.001 | 0.492 | -0.000 | 0.001 | 0.339 |
| Diabetes | 0.126 | 0.021 | <0.001 | 0.097 | 0.021 | <0.001 |
| Metabolic syndrome | 0.139 | 0.012 | <0.001 | 0.117 | 0.012 | <0.001 |
| Cardiovascular disease | 0.238 | 0.030 | <0.001 | 0.205 | 0.030 | <0.001 |
| Weekly alcohol use (grams/week) | -0.0001 | 0.000 | 0.003 | -0.0001 | 0.000 | 0.024 |
| Fraction of alcohol use as wine | -0.101 | 0.019 | <0.001 | -0.070 | 0.019 | 0.002 |
| Smoking status |  |  |  |  |  |  |
| Current | 0.012 | 0.014 | 0.395 | 0.016 | 0.014 | 0.262 |
| Former | 0.020 | 0.016 | 0.216 | 0.031 | 0.016 | 0.062 |
| Never | Ref |  |  | Ref |  |  |
| Exercise |  |  |  |  |  |  |
| At least 2 times a week | Ref |  |  | Ref |  |  |
| 2-4 times a month | -0.061 | 0.014 | <0.001 | -0.044 | 0.014 | 0.002 |
| Less often | -0.030 | 0.017 | 0.078 | -0.008 | 0.017 | 0.647 |
| Low-density lipoprotein cholesterol | -0.004 | 0.006 | 0.497 | -0.003 | 0.006 | 0.659 |
| High-density lipoprotein cholesterol | -0.050 | 0.015 | 0.001 | -0.024 | 0.015 | 0.108 |
| Triglycerides | 0.034 | 0.006 | <0.001 | 0.028 | 0.006 | <0.001 |

^1^ Either measured blood pressure ≥130 (systolic) or ≥85 (diastolic) mmHg or antihypertensive medication use at baseline

Abbreviations: HTA, arterial hypertension; SBP, systolic blood pressure; DBP, diastolic blood pressure; PRS, polygenic risk score; Ref, reference group

Supplementary table 2. P-values for interaction terms between systolic blood pressure polygenic risk score (SBP PRS) or diastolic blood pressure PRS (DBP PRS) and alcohol use, body mass index, waist circumference, diabetes, and prevalent cardiovascular disease in Cox regression analyses with time to incident liver disease as the outcome, and adjustments for age, sex, genotyping chip and the first three principal components of genetic structure.

|  |  |  |
| --- | --- | --- |
|  | SBP PRS,  P-value for interaction | DBP PRS,  P-value for interaction |
| Weekly alcohol use (grams/week) | 0.485 | 0.504 |
| Body mass index | 0.129 | 0.196 |
| Waist circumference | 0.124 | 0.116 |
| Diabetes | 0.746 | 0.675 |
| Cardiovascular disease | 0.561 | 0.934 |
